# Weekly patterns and sociodemographic correlates of adults’ objectively measured physical activity and sedentary behavior

**DOI:** 10.1371/journal.pone.0327662

**Published:** 2025-09-29

**Authors:** Sayaka Kurosawa, Kaori Ishii, Ai Shibata, Mohammad Javad Koohsari, Koichiro Oka

**Affiliations:** 1 College of Sport and Wellness, Rikkyo University, Niiza, Saitama, Japan; 2 Faculty of Sport Sciences, Waseda University, Tokorozawa, Saitama, Japan; 3 Institute of Health and Sport Sciences, University of Tsukuba, Tsukuba, Ibaraki, Japan; 4 School of Advanced Science and Technology, Japan Advanced Institute of Science and Technology, Nomi, Ishikawa, Japan; 5 Institute for Physical Activity and Nutrition, School of Exercise and Nutrition Sciences, Faculty of Health, Deakin University, Geelong, Australia; Universiti Malaya, MALAYSIA

## Abstract

**Objectives:**

As well as the dose-response association of physical activity (PA), its combination with other behaviors and daily patterns are associated with health outcomes. Identifying the prevalence of objectively assessed weekly patterns of PA and sedentary behavior (SB) and sociodemographic correlates may contribute to maximizing beneficial intervention strategies.

**Methods:**

We assessed PA and SB using an accelerometer among 684 participants aged 40–64 years from two Japanese localities. PA and SB combinations were defined as ‘Couch potato’ (low PA and high SB), ‘Light mover’ (low PA and low SB), ‘Sedentary exerciser’ (high PA and high SB), and ‘Busy bee’ (high PA and low SB). Weekly PA patterns were defined as ‘Inactive’ (low PA), ‘Weekend warrior’ (high PA and PA mainly on the weekend), and ‘Regularly active’ (high PA and PA throughout the week). To identify differences in characteristics across the groups, analysis of variance with post-hoc Bonferroni multiple comparison tests was used for continuous variables. For categorical variables, group differences were examined using χ^2^ tests with post-hoc residual analyses.

**Results:**

Regarding PA and SB combinations, ‘Couch potato’ and ‘Busy bee’ groups were approximately one-third each, and ‘Light mover’ and ‘Sedentary exerciser’ were approximately one-fifth-to-sixth. Regarding weekly PA patterns, 16.6% and 38.9% of the participants were categorized as ‘Weekend warrior’ and ‘Regularly active’, respectively. Almost 45% of participants were categorized as ‘Inactive’. The characteristics were defined for each category. For instance, women and participants with lower educational attainment were more likely to be ‘Light mover’. Participants with higher educational attainment were more likely to be ‘Weekend warrior’, whereas age and sex were not significantly associated with weekly PA patterns.

**Conclusions:**

Initiatives to address PA and SB could focus on total amounts and appropriate combinations and patterns depending on the characteristics of the target subgroup.

## Introduction

Physical inactivity and excessive sedentary behavior (SB) have been associated with increased risks of all-cause mortality, cardiovascular diseases, type 2 diabetes mellitus, and certain types of cancer [1,2]. The recent advancement of automatization and computerization in occupational and daily activities has contributed to the growing prevalence of physically inactive and sedentary lifestyles [3]. Therefore, regular monitoring of physical activity (PA) and SB is essential for population health surveillance and the development of effective preventive health strategies.

Previous studies examining the prevalence, correlates, and health impacts of PA and SB have primarily focused on their total amounts [1,2,4]. However, adults may engage in various daily and/or weekly combinations and patterns of PA and SB [5], and these behaviors often do not occur in isolation [2]. From a public health perspective, targeted and tailored intervention should consider not only the prevalence and correlates of such daily combinations of PA and SB but also variations in weekly PA patterns. Notable examples include ‘Sedentary exerciser (high PA and high SB)’ and ‘Weekend warrior (high PA and PA mainly on weekends)’ [6].

Nevertheless, a few studies have investigated the prevalence of PA and SB combinations and weekly PA patterns [7–10]. For example, population-based studies in Sweden (n > 2,000) and the United States (n > 5,000) reported that 21.2%–47.2% of participants were defined as ‘Couch potato (low PA and high SB)’ [7,8]. Also, a pooled analysis of household-based surveillance data from over 60,000 British and Scottish adults found that 3.7%–32.3% were classified as ‘Weekend warrior’ [9,10]. However, these categorizations have varied widely, primarily because most previous studies relied on self-reported measures of PA and SB, which are prone to overestimation or underestimation [11]. A few studies have used triaxial accelerometers to objectively measure interspersed PA and SB with high validity [12].

To date, only two studies have explored differences in limited socioeconomic indicators (i.e., employment status, educational attainment, and household income) across subgroups defined by objectively measured combinations of PA and SB, as well as weekly PA patterns [7,13]. However, no previous research has comprehensively examined how a broader range of demographic, behavioral, and socioeconomic characteristics differ across these subgroups.

Therefore, the present study aimed to address these gaps by (1) estimating the prevalence of PA and SB combinations and weekly PA patterns using accelerometer-derived, objectively measured data, and (2) examining a wider range of demographic, behavioral, and socioeconomic characteristics across the subgroups. This approach offers novel insights into identifying at-risk population profiles and informing the development of targeted interventions to promote PA and reduce SB.

## Materials and methods

This study followed the Strengthening the Reporting of Observational studies in Epidemiology (STROBE) Guidelines.

### Participants and procedure

This cross-sectional survey was conducted between July and December 2013 and from April 2014 to February 2015, as part of a project aiming to identifying the social and urban design characteristics of PA and SB among a sample of middle-aged adults in Japan. The survey design and participant recruitment were previously described [14]. Randomly selected participants (aged 40–64 years) from two Japanese cities (Matsuyama City, Shikoku region; Koto Ward, Tokyo metropolitan area) were included. This study is based on a large-scale survey designed to address multiple research questions. Therefore, no single hypothesis was used to determine the sample size. However, the sample size is considered sufficient to detect meaningful differences among 3–4 groups across multiple dependent variables, based on a G*Power calculation assuming a medium effect size (Cohen’s f = 0.25), a Bonferroni-adjusted alpha level (α = 0.00625), and a statistical power of 0.80, which yields a required sample size of approximately 260–280 participants. A total of 779 participants gave written informed consent and completed an accelerometer and questionnaire survey. This study was approved by the Waseda University Ethics Committee (2012−269) and all methods used in this study were performed in accordance with these guidelines.

### Measurements

#### Objectively measured sedentary behavior and physical activity.

Participants wore a validated tri-axial accelerometer (Active Style Pro HJA-350IT; Omron Healthcare Co. Ltd., Kyoto, Japan) [12,15] for seven consecutive days on their hip during their waking hours, except during water-based activities or contact sports. Details regarding its calibration and validation for middle-aged and older adults have been previously described in studies demonstrating the device’s validity and reliability in measuring PA and SB under free-living conditions [16,17]. Wearing periods and days of the week (weekdays [workdays] and weekends [non-workdays]) were clarified using log diaries and checked along with accelerometer data. Nonwear accelerometer time was defined as intervals of ≥60 consecutive minutes of no activity (≤0.9 metabolic equivalents [METs]) [12], with an allowance for up to 2 minutes of limited movement (≤1.0 METs within these periods) [18]. Days comprising ≥10 h/day wearing time were regarded as valid [18]. Participants with accelerometer data for at least four valid days (including at least one weekend [non-workday]) were included [18]. Two PA and SB measures were designated—total time (min/day) and proportion (% of wear time) of SB, light-intensity physical activity (LPA), and moderate-to-vigorous physical activity (MVPA). The estimated accelerometer intensities were categorized as SB (≤1.5 METs), LPA (>1.5 to <3.0 METs), and MVPA (≥3.0 METs) [19].

#### PA and SB combinations.

PA and SB combinations were classified according to MVPA amounts and SB status [7,20]. In 2023, the official Japanese physical activity guidelines for health promotion were updated. The revised guidelines recommend that Japanese adults aged 18–64 years engage in 60 minutes of MVPA per day without specific consideration of bout duration. Accordingly, participants were categorized into low and high physical activity (PA) groups based on this recommended amount of MVPA. With respect to SB, there is no formal consensus on the threshold above which the risk for important health outcomes significantly increased. However, a meta-analysis showed that sitting for more than 8 h/day significantly increases the risk of all-cause, cardiovascular, and cancer mortality [21]. Also, the potential for using 8 h/day as a measure of excessive SB have been discussed in the development of Canadian and Australian SB public health guidelines [2,22]. Although further research is necessary to validate this threshold, the present study used ≥8 h/day as a proxy indicator of excessive sedentary behavior. The participants were categorized into low- and high-SB groups based on whether their sedentary time was < 8 or ≥8 h/day, respectively.

The following four PA and SB combinations were defined: ‘Couch potato’ (low PA and high SB), ‘Light mover’ (low PA and low SB), ‘Sedentary exerciser’ (high PA and high SB), and ‘Busy bee’ (high PA and low SB) [7,20].

#### Weekly PA patterns.

Weekly PA patterns were classified according to the MVPA amounts and distribution of MVPA across weekdays and weekends [7]. The low- and high-PA groups were categorized based on the recommended amount of MVPA (60 min/day). Distribution of MVPA on weekdays and weekends was based on the weekend/weekday ratio, calculated as the average minutes per day of MVPA on weekends divided by the average minutes per day of MVPA on weekdays. Participants were categorized as ‘PA mainly on weekends’ for weekend/weekday ratios of >1 or ‘PA throughout the week’ for ratios≤1. The amount and weekly distribution values of MVPA were used to define three exclusive weekly pattern groups: ‘Inactive’ (low PA), ‘Weekend warrior’ (high PA and PA mainly on the weekend), and ‘Regularly active’ (high PA and PA throughout the week) [7].

#### Demographic, health behavior, and socioeconomic characteristics.

Data on age and sex of the participants were acquired from residential registries. Participants reported their height, weight, marital status, living condition, education attainment, employment status, household income, smoking status, alcohol consumption, and sleep duration. Body mass index (BMI, kg/m^2^) was calculated using self-reported height and weight. Residential areas were categorized as urban (Koto Ward) or suburban (Matsuyama City).

### Statistical analysis

Of the 779 participants, those with insufficient accelerometer data (*n* = 68) or those with missing or invalid sociodemographic attribute data (*n* = 27) were excluded. After exclusion, 684 participants were included (S1 Fig). Further criteria were set for weekly PA patterns to analyze weekday and weekend patterns. Weekly PA pattern analyses excluded night workers (*n* = 17) because of their different work and sleep patterns, making it challenging to categorize weekdays and weekends. To ensure the representativeness of accelerometer data for weekdays and weekends, participants who did not have three or more weekdays and one or more weekends were excluded (*n* = 76), following previous studies [23]. Finally, a total of 591 participants were included in the weekly PA pattern analysis. Descriptive statistics were reported for PA and SB combinations and weekly PA patterns. To identify differences in characteristics across the groups, one-way analysis of variance (ANOVA) and post-hoc Bonferroni multiple comparison test were used for continuous variables. Effect sizes were presented as partial eta squared (η^2^). The critical partial η^2^ values for determining small, medium and large effects were 0.01, 0.06 and 0.14 respectively [24]. For categorical variables, χ^2^ tests and post-hoc residual analyses were used. Cramer’s V test was performed to calculate the effect size for the χ^2^ tests test, distinguishing 0.06, 0.17, and 0.29 as small, medium, and large effect sizes, respectively [24]. Statistical analyses were performed using IBM SPSS Statistics V.27.0 (IBM Japan Inc., Tokyo, Japan). Statistical significance was set at *p* < 0.05.

## Results

### Participant characteristics

Overall, sociodemographic variable frequencies were similar between the 684 and 591 participants for PA and SB combinations and weekly PA patterns, respectively (S1 Table). Among the participants included in PA and SB combination analyses, the median minutes (% of wear time) of time spent in SB, LPA, and MVPA were 504.5 (54.6%), 355.3 (38.2%), and 63.8 (6.9%), respectively. Among participants included in the weekly PA pattern analysis, the median SB, LPA, and MVPA were 508.4 (55.1%), 352.6 (37.9%), and 63.7 (6.9%), respectively. The proportion of participants who met the recommended amount of MVPA (≥60 min/day) according to the official Japanese physical activity guidelines for health promotion was 55.7% in the PA and SB combination analysis sample and 55.5% in the weekly PA pattern analysis sample. Meanwhile, the proportion of participants who spent in excessive sedentary behavior (≥ 8 h/day) was 56.6% and 58.7% in the respective samples.

### Prevalence of PA and SB combinations and weekly PA patterns

Among PA and SB combinations, the ‘Couch potato’ (*n* = 228, 33.3%) and ‘Busy bee’ (*n* = 220, 32.2%) groups were one-third each, and ‘Light mover’ (*n* = 75, 11.0%) and ‘Sedentary exerciser’ (*n* = 161, 23.5%) groups were less than one-fifth each. Among weekly PA patterns, the ‘Weekend warrior’ and ‘Regularly active’ groups comprised 16.6% (*n* = 98) and 38.9% (*n* = 230) of participants, respectively. The ‘Inactive’ group comprised 44.5% (*n* = 263) participants.

### PA and SB combination and weekly PA pattern characteristics

Table 1 presents PA and SB combination characteristics. ‘Couch potato’ group participants were more likely to have higher frequency of alcohol consumption frequency than other groups. ‘Light mover’ group participants were more likely to be women and living in the suburban residential area and had lower educational attainment. ‘Sedentary exerciser’ group participants were more likely to be living in urban residential areas and have higher educational attainment, household income, and alcohol consumption frequency. ‘Busy bee’ group participants were more likely to be living in a suburban residential area and have lower educational attainment and household income. Only educational attainment, household income, and residential area were distinguished as medium effects.

**Table 1:** Sociodemographic characteristics of combinations of physical activity and sedentary behavior.

	Total (*n* = 684)		Couch potato (Low PA and high SB) (*n* = 228, 33.3%)		Light mover (Low PA and low SB) (*n* = 75, 11.0%)		Sedentary exerciser (High PA and high SB) (*n* = 161, 23.5%)		Busy bee (High PA and low SB) (*n* = 220, 32.2%)		*p*	*η²* *Cramer’s V*
	*n*	(%)	*n*	(%)	*n*	(%)	*n*	(%)	*n*	(%)		
Age, years (mean, (SD))	52.2	(7.1)	52.0	(6.8)	53.0	(7.6)	51.6	(7.3)	52.7	(7.1)	0.375	0.005
Sex: women	417	(61.0)	128	(56.1)	56	(74.7)*	88	(54.7)	145	(65.9)	0.004	0.138
Marital status: married	549	(80.3)	179	(78.5)	65	(86.7)	123	(76.4)	182	(82.7)	0.191	0.083
Living condition												
Living with others	612	(89.5)	203	(89.0)	70	(93.3)	136	(84.5)*	203	(92.3)	0.062	0.104
Living alone	72	(10.5)	25	(11.0)	5	(6.7)	25	(15.5)	17	(7.7)		
Educational attainment												
≤ High school	245	(35.8)	73	(32.0)	35	(46.7)*	39	(24.2)*	98	(44.5)*	<0.001	0.179
≥ 2 years of college	439	(64.2)	155	(68.0)	40	(53.3)	122	(75.8)	122	(55.5)		
Employment status												
Unemployed	123	(18.0)	48	(21.1)	13	(17.3)	25	(15.5)	37	(16.8)	0.505	0.058
Employed	561	(82.0)	180	(78.9)	62	(82.7)	136	(84.5)	183	(83.2)		
Household income												
<5 million yen	308	(45.0)	101	(44.3)	40	(53.3)	47	(29.2)*	120	(54.5)*	<0.001	0.197
≥5 million yen	376	(55.0)	127	(55.7)	35	(46.7)	114	(70.8)	100	(45.5)		
Body mass index, kg/m^2^												
<25	564	(82.5)	180	(78.9)	65	(86.7)	127	(78.9)	192	(87.3)	0.050	0.107
≥25	120	(17.5)	48	(21.1)	10	(13.3)	34	(21.1)	28	(12.7)		
Smoking status: smokers	97	(14.2)	40	(17.5)	13	(17.3)	15	(9.3)	29	(13.2)	0.110	0.094
Alcohol consumption												
≤1–3 times/month	358	(52.3)	132	(57.9)*	44	(58.7)	69	(42.9)*	113	(51.4)	0.019	0.120
≥Once a week	326	(47.7)	96	(42.1)	31	(41.3)	92	(57.1)	107	(48.6)		
Sleep duration												
<7 h/day	563	(82.3)	190	(83.3)	59	(78.7)	141	(87.6)	173	(78.6)	0.114	0.093
≥7 h/day	121	(17.7)	38	(16.7)	16	(21.3)	20	(12.4)	47	(21.4)		
Residential area												
Suburban	351	(51.3)	118	(51.8)	54	(72.0)*	54	(33.5)*	125	(56.8)*	<0.001	0.229
Urban	333	(48.7)	110	(48.2)	21	(28.0)	107	(66.5)	95	(43.2)		

PA = physical activity; SB = sedentary behavior; SD = standard deviation.

Continuous variables: analysis of variance (ANOVA) with post-hoc Bonferroni multiple comparison tests; Categorical variables: χ^2^ tests with post-hoc residual analyses. *Adjusted standardized residual >1.96. Effect sizes: Partial eta squared (η^2^) for ANOVA (small = 0.01, medium = 0.06, large = 0.14); Cramer’s V test for χ^2^ tests (small = 0.06, medium = 0.17, large = 0.29).

Table 2 presents the weekly PA pattern characteristics. ‘Inactive’ group participants were more likely to have a lower frequency of alcohol consumption and live in suburban area than participants of other groups. ‘Weekend warrior’ group participants were more likely to have higher educational attainment and alcohol consumption frequency, and live in urban residential areas. There were no significant proportional differences in any variables between ‘Regularly active’ group and the other groups. All of variables were distinguished as small effect size.

**Table 2:** Sociodemographic characteristics of weekly patterns of physical activity.

	Total (*n* = 591)		Inactive (Low PA) (*n* = 263, 44.5%)		Weekend warrior (High PA and PA mainly on weekends) (*n* = 98, 16.6%)		Regularly active (High PA and PA throughout the week) (*n = *230, 38.9%)		*p*	*η²* *Cramer’s V*
	*n*	(%)	*n*	(%)	*n*	(%)	*n*	(%)		
Age, years (mean, (SD))	52.1	(7.2)	52.0	(7.1)	52.2	(7.3)	52.1	(7.4)	0.958	0.000
Sex: women	365	(61.8)	163	(62.0)	53	(54.1)	149	(64.8)	0.188	0.075
Marital status: married	474	(80.2)	209	(79.5)	76	(77.6)	189	(82.2)	0.581	0.043
Living condition										
Living with others	531	(89.8)	236	(89.7)	83	(84.7)	212	(92.2)	0.121	0.085
Living alone	60	(10.2)	27	(10.3)	15	(15.3)	18	(7.8)		
Educational attainment										
≤High school	204	(34.5)	95	(36.1)	21	(21.4)*	88	(38.3)	0.010	0.124
≥2 years of college	387	(65.5)	168	(63.9)	77	(78.6)	142	(61.7)		
Employment status										
Unemployed	123	(20.8)	61	(23.2)	25	(25.5)	37	(16.1)	0.069	0.095
Employed	468	(79.2)	202	(76.8)	73	(74.5)	193	(83.9)		
Household income										
<5 million yen	261	(44.2)	122	(46.4)	37	(37.8)	102	(44.3)	0.339	0.06
≥5 million yen	330	(55.8)	141	(53.6)	61	(62.2)	128	(55.7)		
Body mass index, kg/m^2^										
<25	488	(82.6)	212	(80.6)	79	(80.6)	197	(85.7)	0.289	0.065
≥25	103	(17.4)	51	(19.4)	19	(19.4)	33	(14.3)		
Smoking status: smokers	81	(13.7)	45	(17.1)	10	(10.2)	26	(11.3)	0.095	0.089
Alcohol consumption										
≤1–3 times/month	309	(52.3)	152	(57.8)*	39	(39.8)*	118	(51.3)	0.009	0.126
≥Once a week	282	(47.7)	111	(42.2)	59	(60.2)	112	(48.7)		
Sleep duration										
<7 h/day	487	(82.4)	218	(82.9)	77	(78.6)	192	(83.5)	0.544	0.045
≥7 h/day	104	(17.6)	45	(17.1)	21	(21.4)	38	(16.5)		
Residential area										
Suburban	301	(50.9)	150	(57.0)*	34	(34.7)*	117	(50.9)	0.001	0.155
Urban	290	(49.1)	113	(43.0)	64	(65.3)	113	(49.1)		

PA = physical activity; SD = standard deviation.

Continuous variables: analysis of variance (ANOVA) with post-hoc Bonferroni multiple comparison tests; Categorical variables: χ^2^ tests with post-hoc residual analyses. *Adjusted standardized residual >1.96. Effect sizes: Partial eta squared (η^2^) for ANOVA (small = 0.01, medium = 0.06, large = 0.14); Cramer’s V test for χ^2^ tests (small = 0.06, medium = 0.17, large = 0.29).

## Discussion

Categorizing populations by their similar PA and SB characteristics is necessary to develop more persuasive and feasible intervention strategies than a one-size-fits-all approach. This study identified the prevalence of objectively assessed PA and SB combinations and weekly PA patterns and their characteristics.

Although comparisons are difficult owing to the use of inconsistent behavior assessment tools, previous population-based studies conducted with large-scale samples exceeding one thousand participants found that objectively assessed ‘Couch potato’ and ‘Inactive’ participants were approximately 21%–47% and 30%–33%, respectively [7,8,10]. In this study, objectively assessed PA and SB using the tri-axial accelerometer confirmed the high prevalence of the most high-risk subgroups (‘Couch potato’ and ‘Inactive’), consistent with previous estimates [7,8,10]. Given that the adverse health outcome effects of physical inactivity may be greater for those who are more sedentary [25], ordinal intervention strategies (reducing total SB and promoting total PA) based on the characteristics of those with ‘Couch potato’ and ‘Inactive’ patterns may greatly impact intervention effects.

This study also found that a noticeable proportion of participants were categorized as ‘Sedentary exerciser’, ‘Light mover’, and ‘Weekend warrior’, which may have varying daily behavioral routines [26]. These results suggest that the opportunities and modifiable means for engaging in LPA and MVPA accompanying the breakup of SB may vary depending on a group’s characteristics or status. The PA and SB combination and weekly PA patterns might account for target segments that would have been hidden if only the total PA amount had been evaluated. Intervention strategies for providing counseling and information tailored to each of the three groups’ behavioral patterns may expand intervention effectiveness.

This study found that sociodemographic characteristics differed by group. Intrapersonal characteristics (e.g., sex, family situation, and health behaviors), as well as social and environmental characteristics, might largely influence the PA and SB combinations and weekly PA patterns distinguishing each group. Previous studies have shown that PA and SB are related to socioeconomic status and environment [4], which are partly but closely associated with residential areas. Approaches tailored to modifiable intrapersonal characteristics and social and environmental factors must be developed and tested. Although the present study did not examine the underlying mechanisms behind these group differences, future research should investigate potential psychosocial and contextual factors that may explain why specific subgroups, like individuals who have higher educational attainment, are more likely to exhibit certain behavioral patterns such as the “Weekend Warrior” profile. Understanding these mechanisms will be crucial for designing more effective interventions.

To our knowledge, this is the first report of objectively assessed PA and SB combinations and weekly patterns and their various characteristics. However, this study has some limitations. First, the study’s cross-sectional design prevents causal relationship determination. A longitudinal design would be more suitable for exploring and identifying causal links between PA, SB, and sociodemographic variables. Second, the hip-worn accelerometer used in this study could not measure water-based activities, and leg and arm movement, nor accurately differentiate between sitting and static standing postures. Third, the low response rate in this study may have resulted in selection bias. Fourth, as this study was conducted among Japanese adults, cultural norms and lifestyle factors unique to the Japanese context may have influenced the observed patterns. Therefore, caution should be taken when generalizing the findings to populations in other cultural settings. Fifth, the data were collected between 2013 and 2015, prior to major societal shifts such as the COVID-19 pandemic, which has profoundly affected PA and SB patterns worldwide. In particular, changes in work styles (e.g., increased remote work) and urban mobility may have altered behavioral trends. Thus, future research using more recent post-pandemic data is necessary to confirm the present findings and assess potential changes in population-level PA and SB patterns. Lastly, this study selected Japanese public health recommendation for physical activity as criteria of MVPA. However, it should be noted that the WHO’s updated recommendations in 2020 differ from the thresholds and criteria used in our study. Therefore, caution is needed when interpreting our results in the context of current international guidelines.

## Conclusion

This study determined the prevalence of distinct category groups in PA and SB combinations and weekly patterns, potentially targeting behavioral interventions. Among PA and SB combinations, ‘Couch potato’ and ‘Busy bee’ each comprised approximately one-third, and ‘Light mover’ and ‘Sedentary exerciser’ comprised approximately one-fifth-to-sixth of the middle-aged adults. Among weekly PA patterns, ‘Weekend warrior’ and ‘Regularly active’ comprised 16.6% and 38.9% of participants, respectively. We showed that sex, educational attainment, household income, alcohol consumption, and residential area were differently associated with PA and SB combinations and weekly patterns. Although initiatives to address adult PA and SB could focus on promoting PA and reducing SB, considering their combinations and weekly patterns and characteristics may improve the feasibility and effectiveness of interventions.

## Supporting information

S1 Fig Flowchart of participant recruitment. (TIFF)

S1 Table Basic characteristics of study participants. (DOCX)
