# Supplementary figures and images for: Weekly patterns and sociodemographic correlates of adults’ objectively measured physical activity and sedentary behavior
Source: PLoS One. 2025 Sep 29;20(9):e0327662. doi: 10.1371/journal.pone.0327662 (PMC12478925; doi:10.1371/journal.pone.0327662)

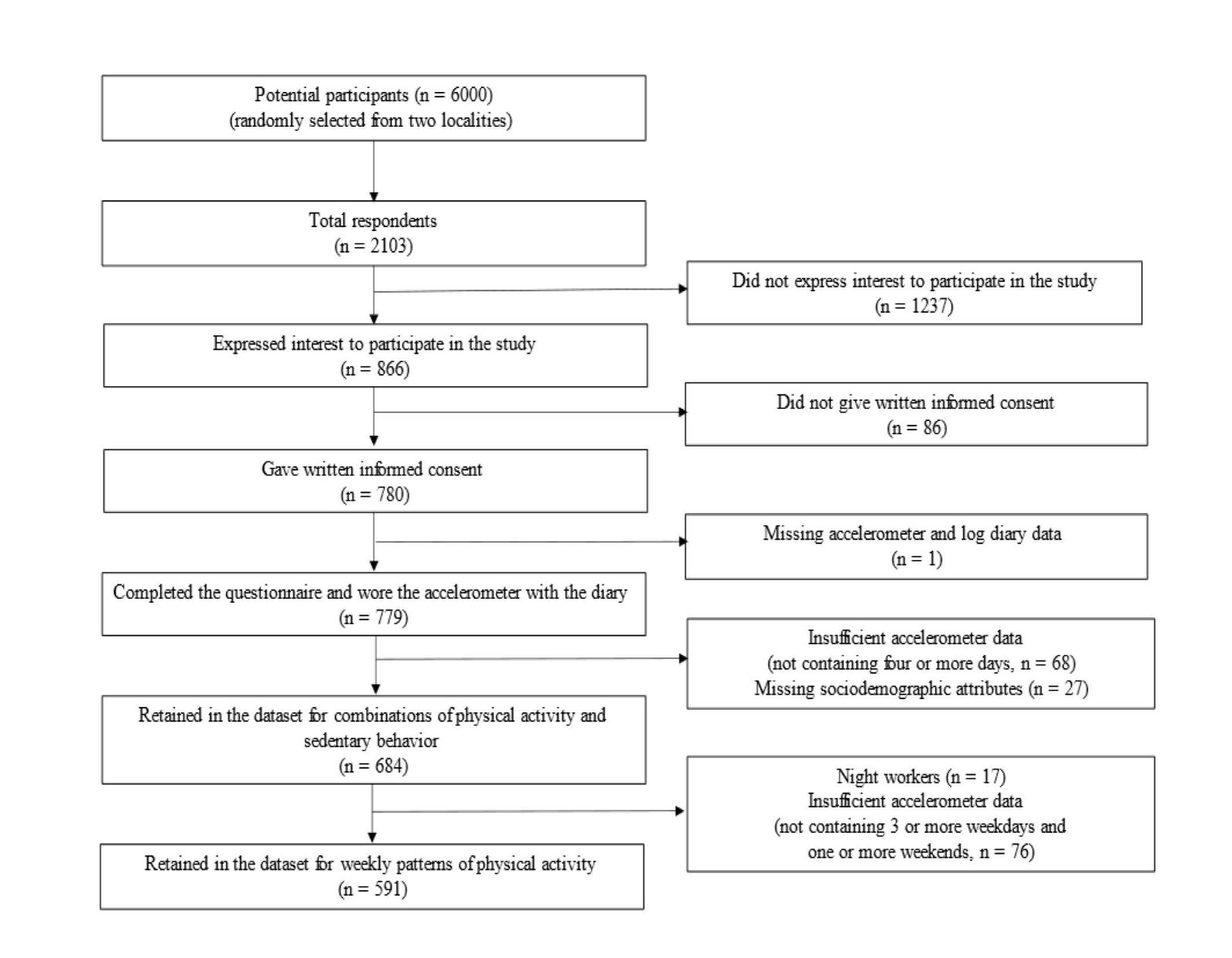

Supplement: S1 Fig — (TIFF) [file pone.0327662.s001.tiff]
