# Supplementary material for: Weekly patterns and sociodemographic correlates of adults’ objectively measured physical activity and sedentary behavior
Source: PLoS One. 2025 Sep 29;20(9):e0327662. doi: 10.1371/journal.pone.0327662 (PMC12478925; doi:10.1371/journal.pone.0327662)
Supplement: S1 Table — (DOCX) [file pone.0327662.s002.docx]

**S1 Table.** Basic characteristics of study participants

|  | Combinations of physical activity and sedentary behavior  *n* (%) | Weekly patterns of physical activity  *n* (%) |
| --- | --- | --- |
| Age, years (mean, (SD)) | 52.2 (7.1) | 52.1 (7.2) |
| Sex: women | 417 (61.0) | 365 (61.8) |
| Marital status: married | 549 (80.3) | 474 (80.2) |
| Living condition: living with others | 612 (89.5) | 531 (89.8) |
| Educational attainment |  |  |
| ≤High school | 245 (35.8) | 204 (34.5) |
| ≥2 years of college | 439 (64.2) | 387 (65.5) |
| Employment status: employed | 561 (82.0) | 468 (79.2) |
| Household income |  |  |
| <5 million yen | 308 (45.0) | 261 (44.2) |
| ≥5 million yen | 376 (55.0) | 330 (55.8) |
| BMI (mean±SD) | 22.4 (3.2) | 22.4 (3.3) |
| Smoking status: smokers | 97 (14.2) | 81 (13.7) |
| Alcohol consumption |  |  |
| ≤1–3 times/month | 358 (52.3) | 309 (52.3) |
| ≥1 time/week | 326 (47.7) | 282 (47.7) |
| Sleep duration |  |  |
| <7 h/day | 563 (82.3) | 487 (82.4) |
| ≥7 h/day | 121 (17.7) | 104 (17.6) |
| Residential area |  |  |
| Suburban | 351 (51.3) | 301 (50.9) |
| Urban | 333 (48.7) | 290 (49.1) |

SD, standard deviation; BMI, body mass index
